# A Cost-Effective, Kit-Free Method for Extracting Nuclear and Mitochondrial DNA From Low-Abundance Cells in Urine Samples

**DOI:** 10.7759/cureus.116804

**Published:** 2026-09-23

**Authors:** Yoshitaka Yamaguchi, Koichi Yamaguchi, Hiroshi Suzuki, Junko Nomoto, Hironori Katayama

**Affiliations:** 1 Department of Medical Technology and Sciences, School of Health Sciences at Narita, International University of Health and Welfare, Narita, JPN; 2 Department of Medical Technology, Faculty of Medical and Health Sciences, Tsukuba International University, Tsukuba, JPN; 3 Department of Clinical Laboratory Science, Faculty of Health Sciences, Japan Healthcare University, Sapporo, JPN; 4 Department of Medical Laboratory Sciences, Graduate School, International University of Health and Welfare, Narita, JPN

**Keywords:** cost-effective molecular diagnostics, kit-free dna extraction, low-biomass specimens, mitochondrial dna, urine liquid biopsy

## Abstract

This study evaluates a simplified, cost-effective DNA extraction protocol using only 10% sodium chloride (NaCl) and Proteinase K (ProK) to isolate both nuclear DNA (nuDNA) and mitochondrial DNA (mtDNA) from low-abundance cells in human urine, providing an open-source alternative to expensive commercial kits. Urine samples from six healthy participants were centrifuged, washed with phosphate-buffered saline, lysed with the NaCl/ProK mixture at 60°C overnight, and recovered via ethanol precipitation. To validate downstream applicability, polymerase chain reaction (PCR) amplification was performed targeting the nuclear *FGFR3* (423 bp) and the mitochondrial D-loop regions (HV-1: 634 bp, HV-2: 520 bp), followed by agarose gel electrophoresis and Sanger sequencing. The extracted DNA concentrations ranged from undetected to 146.0 μg/mL. PCR amplification of the nuclear *FGFR3* succeeded in five out of six samples (83.3%), while the mitochondrial regions (HV-1 and HV-2) achieved a 100% success rate, yielding robust bands even in the sample with undetectable initial genomic DNA. Sanger sequencing and Basic Local Alignment Search Tool search confirmed 100% (*FGFR3*), 99.4% (HV-1), and 97.8% (HV-2) homology with reference sequences (National Center for Biotechnology Information: NC_012920.1). In conclusion, sequence-ready nuDNA and mtDNA can be successfully extracted using this minimal-reagent, kit-free method. The significantly higher sensitivity of mtDNA highlights its potential for non-invasive genetic diagnostics and point-of-care liquid biopsy in low-biomass specimens.

## Introduction

Liquid biopsy has emerged as a revolutionary paradigm in modern clinical oncology, prenatal screening, and genetic monitoring, offering a minimally invasive alternative to traditional tissue biopsies [1]. Among various biological fluids, urine represents an exceptionally attractive source for liquid biopsy because its collection is entirely non-invasive, cost-effective, can be performed without specialized medical personnel, and provides large volumes continuously [2]. For urological malignancies, particularly bladder cancer and prostate cancer, urine is in direct contact with the tumor microenvironment, meaning it contains exfoliated tumor cells, cell-free DNA (cfDNA), and extracellular vesicles that directly mirror the genetic landscape of the malignancy [3,4].

The genetic alterations associated with bladder cancer pathogenesis frequently involve specific somatic mutations, such as those in the fibroblast growth factor receptor 3 (*FGFR3*) gene [5], as well as chromosomal abnormalities such as 9q loss of heterozygosity (LOH) [6]. Detecting these nuclear DNA (nuDNA) alterations in urine has demonstrated high clinical utility for early diagnosis and post-treatment surveillance, significantly reducing the need for invasive transurethral cystoscopy [7]. However, because the absolute cell count and nucleic acid concentration in the urine of healthy individuals or early-stage patients are often extremely scarce, achieving reproducible nuDNA amplification remains a profound technical challenge [4].

To complement the limitations of low-abundance nuDNA, human mitochondrial DNA (mtDNA) has gained substantial traction as a vital biomarker [8,9]. Containing a 16,569 bp circular genome with a highly variable control region (D-loop), mtDNA exists in hundreds to thousands of copies per cell [10,11]. This high copy number per cell inherently provides a significantly superior analytical sensitivity compared to the diploid nuclear genome, making mtDNA an ideal tracer for forensic identification [12], population genetics [13], and low-biomass disease tracking in urine specimens [14].

Despite the clear clinical utility of urine-based genetic testing, its widespread global implementation, especially in resource-limited laboratories and decentralized testing facilities, is severely bottlenecked by upfront sample processing (nucleic acid extraction, concentration, filtration, etc.). Standard diagnostic laboratories heavily rely on commercial spin-column or magnetic bead-based extraction kits. While these commercial solutions are robust and automated, they require expensive proprietary reagents, specialized centrifuges, and structured cold-chain logistics, which dramatically inflate the cost per test. Furthermore, potential nucleic acid loss during column or magnetic bead purification procedures has been noted as a general concern in handling low-biomass specimens [15,16].

Additionally, commercial kits often utilize chaotropic guanidinium salts or hazardous organic solvents, generating chemical waste that restricts their use outside of centralized facilities [16]. Therefore, establishing a rudimentary, open-source, yet highly efficient extraction protocol that streamlines sample handling by reducing tube-to-tube transfers and maximizing target recovery using basic, universally accessible, and stable laboratory reagents is highly desirable to democratize non-invasive molecular diagnostics.

To address this technological gap, this study evaluates and demonstrates a simplified, cost-effective DNA extraction protocol that excludes all commercial kit components. By utilizing a minimalist system consisting exclusively of 10% sodium chloride (NaCl) and Proteinase K (ProK) enzymatic digestion combined with standard ethanol precipitation, we succeeded in isolating sequence-ready nuclear and mitochondrial DNA from small cell fractions in human urine. This protocol simplifies sample handling by confining the enzymatic lysis and precipitation steps to a simplified container system. This study validates the workflow’s efficacy through downstream polymerase chain reaction (PCR) amplification, agarose gel visualization, and direct Sanger sequencing, offering an accessible blueprint for low-cost molecular workflows. Furthermore, this study focuses on a technique for extracting DNA from urinary cells, and further research is needed regarding techniques for recovering urinary cfDNA.

This article was previously presented as an oral presentation at the 61st Metropolitan and Kanto-Koshinetsu Joint Conference of Medical Technology on October 10-11, 2025.

## Technical report

Methodology

Sample Collection and Ethical Considerations

Urine samples were obtained from six healthy individuals in their 20s. This study was reviewed and deemed exempt from full review by the Institutional Review Board of the International University of Health and Welfare (approval number: 24-Im-008-2, approval date: 30 September 2025). Written informed consent was obtained from all individual participants included in the study.

DNA Extraction Protocol

Morning urine samples (50-200 mL) were collected from six participants. The urine samples were transferred into 50 mL tubes and centrifuged at 190 × g for 10 minutes at room temperature to pellet the cells (Model 4000, KUBOTA, Tokyo, Japan). The supernatant was aspirated, leaving approximately 2 mL to avoid disturbing the invisible cell pellet. The pellet was washed twice with 30 mL of phosphate-buffered saline (PBS) and centrifuged under the same conditions. The remaining supernatant was removed as completely as possible using a micropipette. For cell lysis and DNA preservation, 1 mL of 10% NaCl solution prepared with UltraPure DNase/RNase-Free Distilled Water (Invitrogen, Thermo Fisher Scientific, Waltham, MA, USA) and 1 μL of ProK (20 mg/mL; Promega, Madison, WI, USA) were added to the cell pellet, followed by overnight incubation at 60°C (Figure 1).

**Figure 1:**
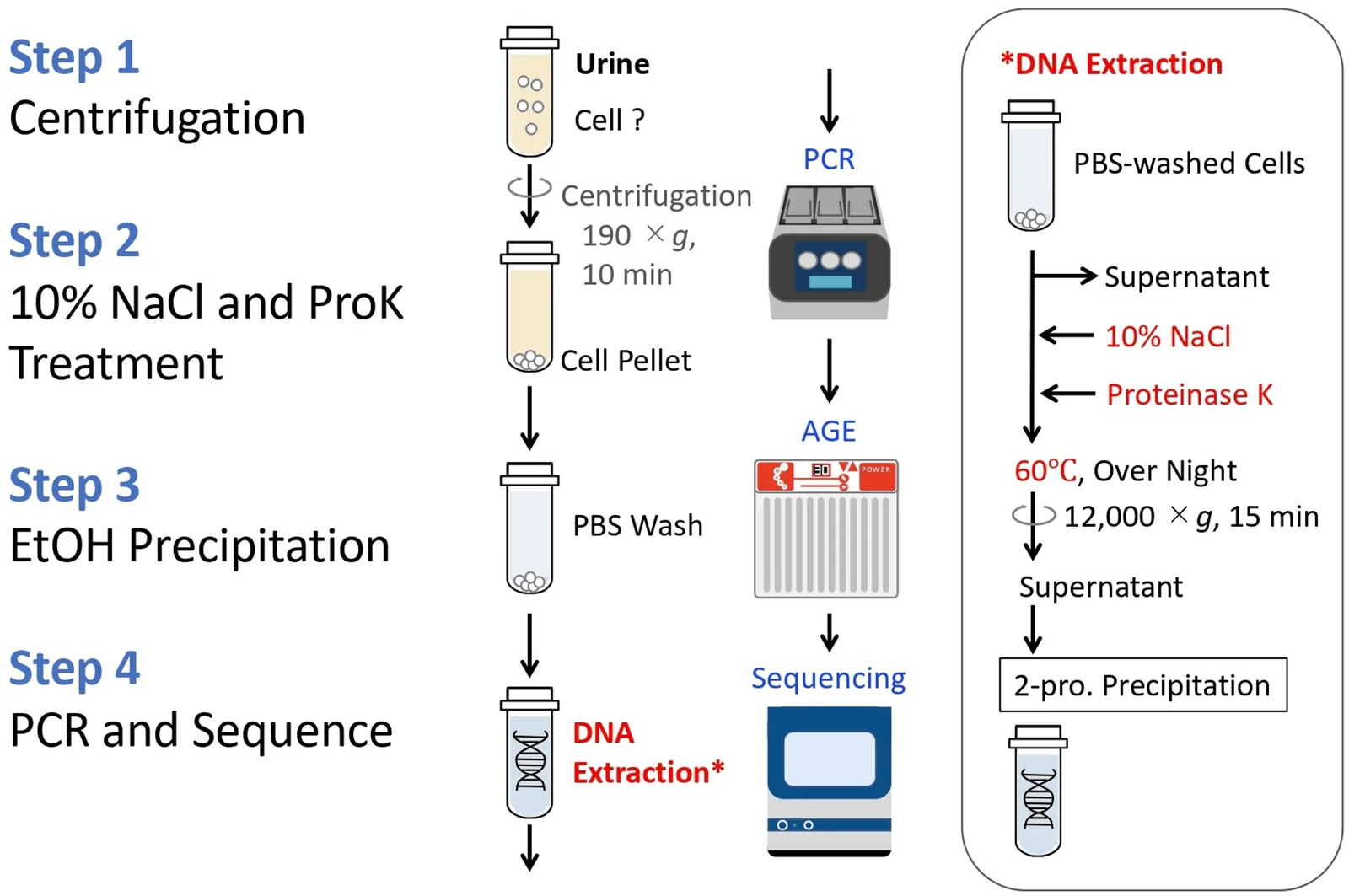
Workflow of the simplified DNA extraction and downstream genetic analysis from urine samples. The protocol comprises four steps: Step 1: Centrifugation of urine to obtain a cell pellet, followed by a PBS wash to remove PCR inhibitors. Step 2: Cell lysis and protein digestion overnight at 60℃ using only 10% NaCl and ProK. Step 3: Recovery of purified genomic DNA via isopropanol precipitation. Step 4: Downstream validation using PCR, AGE, and Sanger sequencing. Image credits: Created manually by Yoshitaka Yamaguchi using Microsoft PowerPoint for Mac. No artificial intelligence tools were used to generate or modify this image. PBS = phosphate-buffered saline; PCR = polymerase chain reaction; ProK = Proteinase K; AGE = agarose gel electrophoresis

Following enzymatic digestion and heat treatment, 700 μL of isopropanol was added to the samples, mixed, and incubated at room temperature for 10 minutes (isopropanol precipitation). The mixture was then centrifuged at 12,000 × g for 15 minutes using a micro refrigerated centrifuge (Model 3740, KUBOTA, Tokyo, Japan). The supernatant was removed by decantation, and the DNA pellet was rinsed with 1 mL of cold 70% ethanol. After removing the ethanol, the DNA pellet was dissolved in 100 μL of 10 mM Tris-HCl (pH 8.0, containing 1 mM ethylenediaminetetraacetic acid (EDTA)) buffer and left overnight in a refrigerator at 4°C (Figure 1, EtOH Precipitation). The concentration of the extracted genomic DNA (μg/mL) was measured for all six samples using a Qubit 3.0 fluorometer (Invitrogen, Thermo Fisher Scientific, Waltham, MA, USA).

Polymerase Chain Reaction Amplification

To verify the compatibility of the extracted DNA with downstream molecular diagnostics, PCR was performed targeting both nuclear and mitochondrial genomic loci using specific primers detailed in Table 1 [5]. Gene-specific primers were designed using the Primer3 web-based tool and synthesized by Integrated DNA Technologies (IDT, Coralville, IA, USA). Detailed information on the primers is presented in Table 1.

**Table 1:** Characteristics of PCR primers targeting the nuclear FGFR3 gene and mitochondrial D-loop regions. Created by the authors based on original study data. PCR = polymerase chain reaction

Name	Sequence (5’>3’)	Tm	Product Size
*FGFR3*-F	GGTGACACTCTTCGTCCTTACGA	64℃	423 bp
*FGFR3*-R	TCATCATCTCCATCTCAGACACCAG	65℃	
HV1-F	TACTCAAATGGGCCTGTCCTTGTAG	60℃	636 bp
HV1-R	GGAACCAGATGTCGGATACAGTTCAC	61℃	
HV2-F	GTGAACTGTATCCGACATCTGGTTCC	61℃	520 bp
HV2-R	GTGACTGTTAAAAGTGCATACCGCC	60℃	

PCR was performed using AmpliTaq Gold 360 DNA Polymerase (Thermo Fisher Scientific, Waltham, MA, USA). The reaction mixture was prepared in a total volume of 10 μL, containing 5 μL of AmpliTaq Gold 360 Master Mix, 0.3 μL each of 10 μM forward and reverse primers (final concentration: 0.3 μM), 1 μL of the extracted DNA template, and 3.4 μL of DNase/RNase-Free Water.

The thermal cycling profile was executed as follows: an initial denaturation step at 95°C for five minutes; followed by 40 cycles of denaturation at 95°C for 20 seconds, annealing at 62°C for *FGFR3* (Figure 2) or 60°C for HV-1 and HV-2 (Figure 3) for 20 seconds, and extension at 72°C for 20 seconds; and a final elongation step at 72°C for five minutes. Following amplification, the PCR products were separated and visualized using agarose gel electrophoresis (i-MyRun; Cosmo Bio Co., Ltd., Tokyo, Japan) alongside a 50 bp DNA ladder (Nippon Genetics Co., Ltd., Tokyo, Japan).

**Figure 2:**
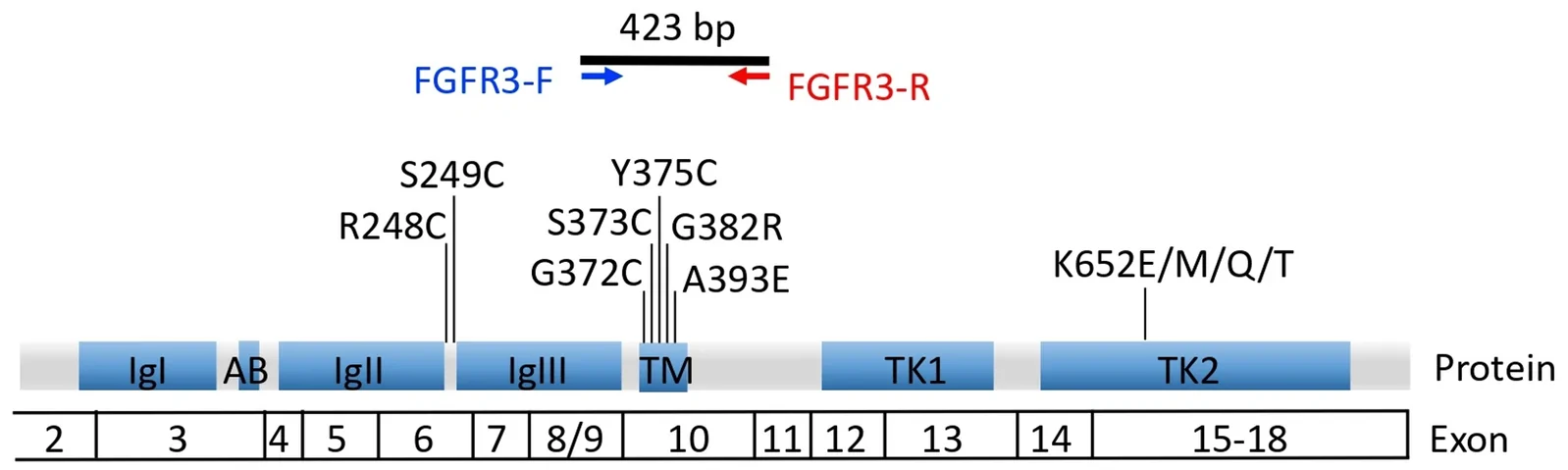
Schematic representation of the FGFR3 gene structure, protein domains, and primer positions. The diagram illustrates the correlation between exons (bottom) and corresponding protein domains (middle), including immunoglobulin-like domains (IgI, IgII, IgIII), acid box (AB), transmembrane domain (TM), and split tyrosine kinase domains (TK1, TK2). Major somatic mutation sites associated with bladder cancer (e.g., R248C, S249C, G372C, S373C, Y375C, G382R, A393E, K652E/M/Q/T) are indicated. The forward (FGFR3-F) and reverse (FGFR3-R) primers are positioned to amplify a 423 bp fragment spanning across the IgIII, TM, and adjacent regions. Figure modified from Margaret A. Knowles (2020) [5] under the terms of the Creative Commons Attribution License (CC BY-NC 4.0).

**Figure 3:**
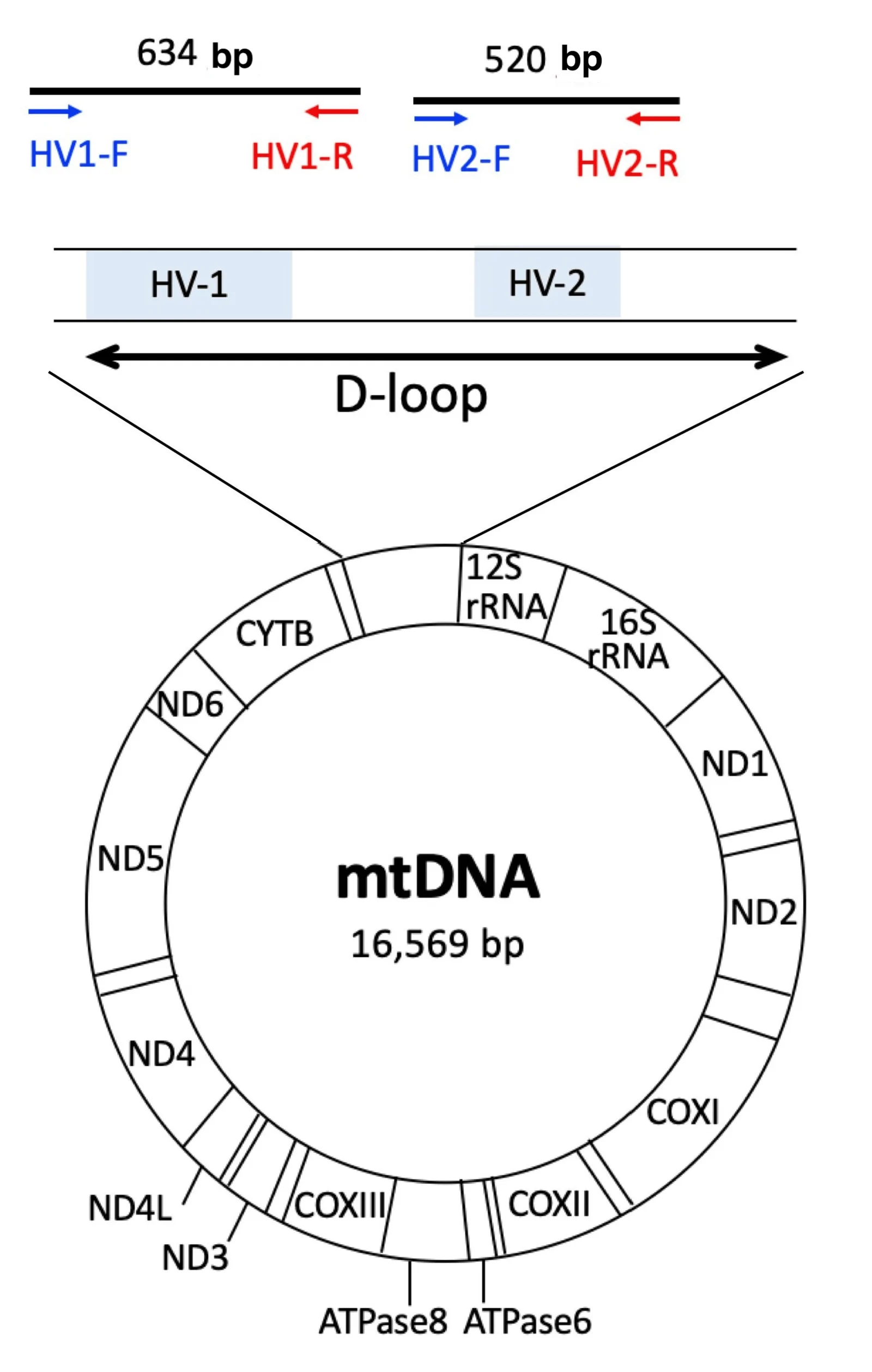
Schematic structure of human mitochondrial DNA (mtDNA) and primer positions within the D-loop region. The lower panel shows the 16,569 bp circular mitochondrial genome including genes for NADH dehydrogenase (ND), cytochrome C reductase (CYTB), cytochrome C oxidase (COX), ATP synthase (ATPase), and ribosomal RNA (rRNA). The upper panel illustrates the hypervariable regions (HV-1 and HV-2) inside the non-coding D-loop control region. The primer pairs are designed to target and amplify 636 bp fragment for HV-1 and 520 bp fragment for HV-2, respectively. The International University of Health and Welfare gratefully acknowledges the U.S. Department of Justice, Office of Justice Programs, National Institute of Justice, for allowing us to reproduce, in part or in whole, the publication “Non-STR DNA Markers: SNPs, Y-STRs, LCN and mtDNA (Mitochondrial DNA Applications),” which was used as a reference to create this figure. The opinions, findings, and conclusions or recommendations expressed in this study are those of the authors and do not necessarily represent the official position or policies of the U.S. Department of Justice. Created by Yoshitaka Yamaguchi based on information from reference [17].

Sequencing and Homology Analysis

To determine the nucleotide sequence of the successfully amplified PCR fragments, the amplicons were first treated with ExoSAP-IT Express PCR Product Cleanup Reagent (Thermo Fisher Scientific, Waltham, MA, USA) to remove enzymatic residues and excess primers [7]. Following enzymatic cleanup, cycle sequencing reactions were performed using the BigDye Terminator v3.1 Cycle Sequencing Kit (Thermo Fisher Scientific, Waltham, MA, USA). The sequencing reaction products were then purified and cleaned using the BigDye XTerminator Purification Kit (Thermo Fisher Scientific, Waltham, MA, USA) to eliminate unincorporated dye terminators and salts. The purified DNA fragments were subsequently analyzed and sequenced on a 3500xL Genetic Analyzer (Thermo Fisher Scientific, Waltham, MA, USA). Finally, the decoded nucleotide sequences were subjected to a homology search and cross-verified against the National Center for Biotechnology Information (NCBI) database using the Basic Local Alignment Search Tool (BLAST).

Results

DNA Yield and Purity

The genomic DNA concentrations obtained via the NaCl/ProK extraction method revealed substantial inter-individual variation, reflective of the naturally fluctuating cell numbers found in physiological urine samples (Table 2). Although one sample was not detected by the Qubit 3.0 Fluorometer, 0.3-146.0 ng of DNA was extracted from urinary cells. DNA was extracted from the urine of two men and four women in their 20s. As the result for one male was “ND” (not detected), the average yield was 5.0 ng for the men and 1,419.4 ng for the women.

**Table 2:** Quantitative yield of genomic DNA extracted from urine samples. Data represent the urine volume, genomic DNA concentration, yield per unit volume, and spectrophotometric purity ratios A260/A280 and A260/A230 of six healthy participants. The urine volume indicates an approximate amount. ND = not detected

Sample	Sex	Urine volume (mL)	DNA concentration (μg/mL)	DNA yield (ng/mL urine)	A260/A280	A260/A230
1	Female	200	146.0	730.0	1.58	0.86
2	Male	60	0.3	5.0	1.58	0.83
3	Female	110	32.2	292.7	1.85	2.57
4	Female	60	0.7	11.7	1.62	1.82
5	Female	80	30.8	385.0	1.71	0.91
6	Male	50	ND	ND	1.59	0.85

The quantitative yields and optical purity ratios A260/A280 and A260/A230 of the extracted genomic DNA are summarized in Table 2. The DNA concentration and overall yield exhibited substantial inter-individual variations, ranging from non-detectable (ND) to 146.0 μg/mL (yield: 730.0 ng/mL urine). Female participants (Samples 1, 3, and 5) consistently demonstrated markedly higher DNA yields (292.7-730.0 ng/mL urine) compared to male participants (Samples 2 and 6; 0-5.0 ng/mL urine).

In terms of purity, the A260/A280 ratio averaged approximately 1.65, with Sample 3 achieving an optimal ratio of 1.85 and Sample 5 yielding 1.71. Conversely, Samples 1, 2, and 6 showed slightly lower values (1.58-1.59). The A260/A230 ratios demonstrated wider fluctuation; Samples 3 and 4 achieved high ratios of 2.57 and 1.82, respectively, whereas Samples 1, 2, 5, and 6 exhibited lower ratios (0.83-0.91). Notably, Sample 6 yielded an A260/A230 reading of 0.85 despite the genomic DNA concentration being below the fluorometric/spectrophotometric limit of detection (ND).

Polymerase Chain Reaction Amplification Success

Amplicons from the nuclear and mitochondrial loci were evaluated using 2% agarose gel electrophoresis. For nuDNA (*FGFR3*), a distinct band at 423 bp was detected in five out of the six samples (Samples 1-5), yielding an overall nuclear success rate of 83.3%. As expected from the fluorometric quantification, Sample 6 did not yield a visible band, matching the negative control (N). In contrast, mtDNA (HV-1 and HV-2) showed a 100% success rate, with clear, distinct bands matching the expected target sizes (636 bp for HV-1 and 520 bp for HV-2) successfully captured in all six samples. Strikingly, Sample 6 exhibited robust amplification for both mitochondrial fragments despite having an initial total genomic DNA reading below the fluorometric limit of detection (Figure 4).

**Figure 4:**
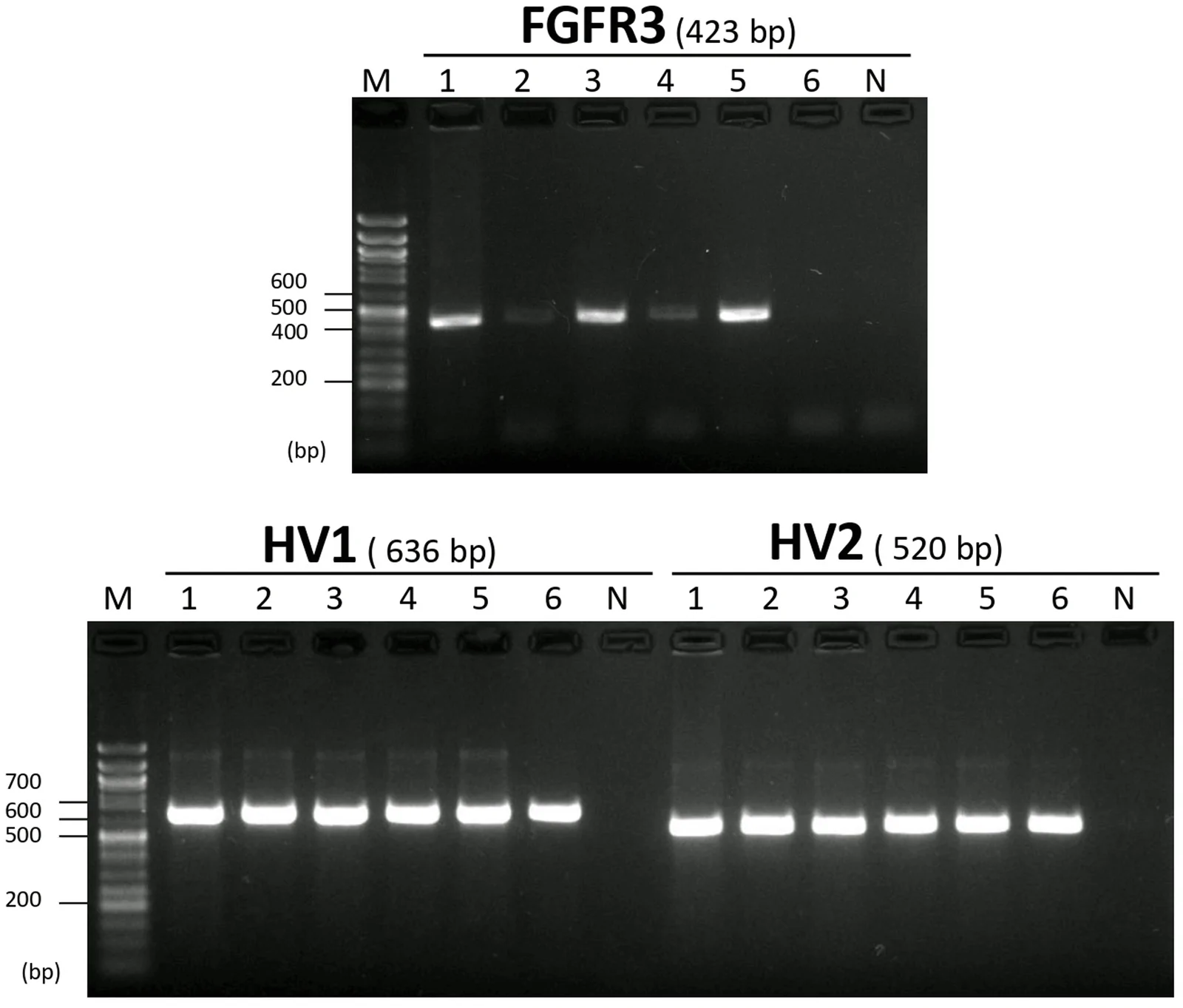
PCR analysis of the extracted nuclear and mitochondrial DNA. The gels show amplified fragments of the nuclear FGFR3 (423 bp) and mitochondrial D-loop regions (HV1, 636 bp; HV2, 520 bp). Lanes are designated as follows: M: 50 bp Ladder marker (bp); Lanes 1–6: urine DNA samples from healthy participants 1 to 6; N: negative control (no template). Distinct bands represent successful target gene amplification. PCR = polymerase chain reaction

Sequencing and Alignment Analysis

The upper panel of Figure 5 shows the sequencing results of the PCR products amplified from the mtDNA HV-1 region. The region highlighted in blue corresponds to the HV-1 region, which was detected with clear single peaks across all samples. The lower panel displays the alignment analysis between the reference sequence of the mtDNA HV-1 region (NCBI, NC_012920.1) and the sequenced samples. A single-nucleotide mismatch with the reference sequence was observed at position 184 (Reference sequence: C, Sample: T).

**Figure 5:**
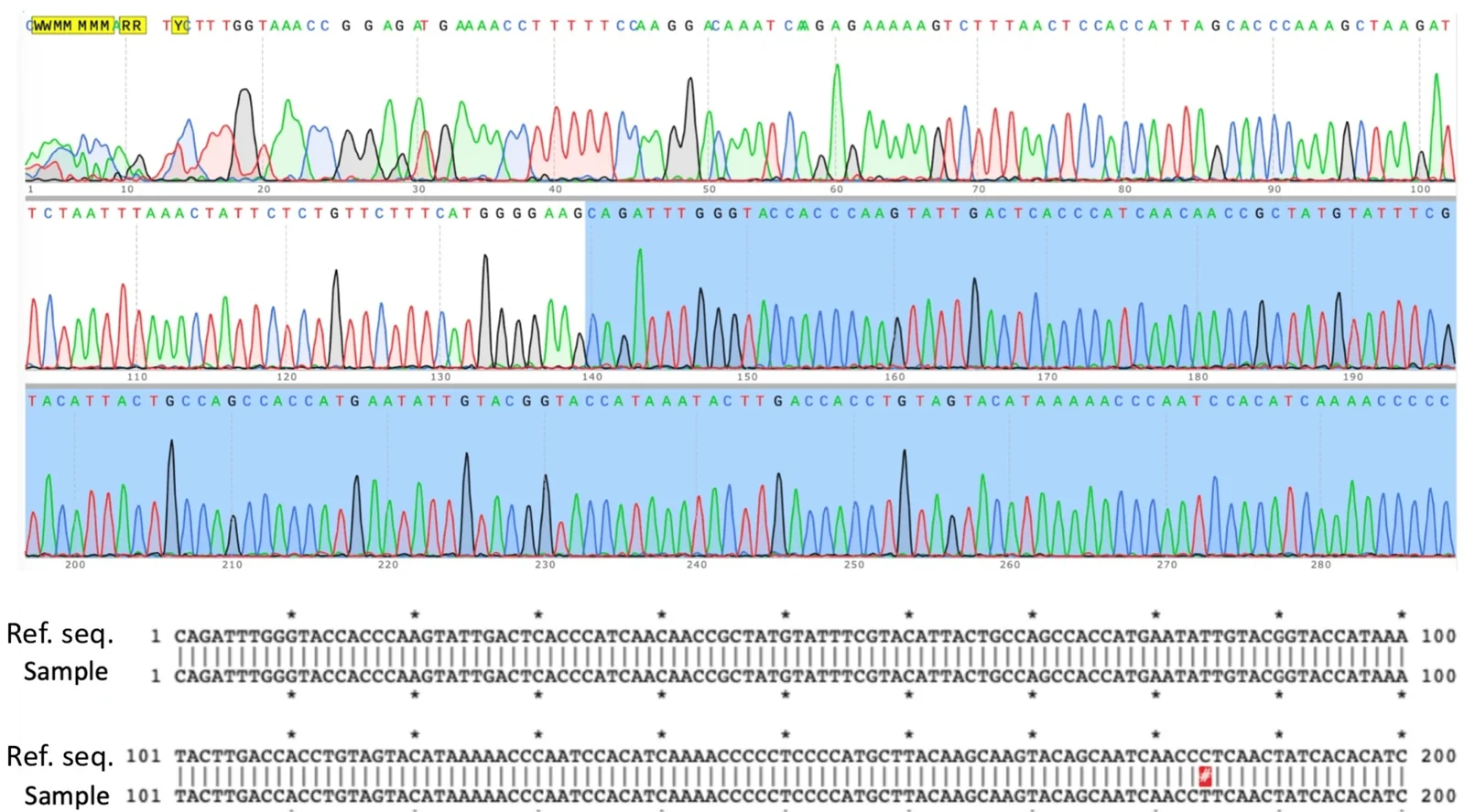
Sequencing and alignment analysis of the HV-1 region. Upper: Sanger sequencing chromatogram of the PCR product covering the HV-1 region. The blue highlighted area indicates the HV-1 region, showing clear single peaks in the electropherogram. Lower: Alignment of the sequenced sample with the reference human mtDNA sequence (NCBI Accession No. NC_012920.1) in the HV-1 region. A single nucleotide mismatch was identified at position 184 (reference sequence: C, sample: T). PCR = polymerase chain reaction

Sequence Homology

Sanger sequencing chromatograms demonstrated high-quality, clean trace peaks across the target segments (data not shown). A BLAST search for the *FGFR3* amplicon demonstrated a 100% identity match (50/50 base pairs aligned with zero gaps) with *Homo sapiens* *FGFR3*, RefSeqGene (NG_012632.1) located on chromosome 4, while Sanger sequencing and a BLAST search for the mitochondrial control region confirmed 99.4% homology for HV-1 and 97.8% homology for HV-2 with the reference sequences.

## Discussion

This study demonstrates that a basic approach utilizing only 10% NaCl and ProK is remarkably efficient for extracting sequencing-ready DNA from urinary cells, even when cell counts are extremely low. Commercial DNA extraction kits generally rely on chaotropic salts, spin columns, or automated magnetic beads to bind and retain DNA while washing away impurities. While these kits are convenient, they present challenges regarding high costs [16]. While commercial kits provide convenient workflows, concerns regarding reagent costs and procedural loss during column or bead separation have been reported in low-biomass settings [15,16,18]. In contrast, our open-tube method combines high-salt conditions (10% NaCl) and ProK-mediated proteolysis with heat treatment at 60°C to achieve cell membrane lysis. By utilizing a direct alcohol precipitation strategy without solid-phase binding matrices, this method provides a simple, kit-free alternative that successfully recovers amplifiable DNA using widely available reagents.

Spectrophotometric purity profiles revealed distinct characteristics inherent to the high-salt extraction system. The A260/A280 ratio serves as a standard metric for protein contamination, where values between 1.8 and 2.0 represent high-purity DNA. In our cohort, the average A260/A280 ratio was approximately 1.65, suggesting minor co-precipitation of cellular proteins or hydrophobic impurities. Prolonging the ProK digestion time or optimizing the enzyme concentration could further enhance protein degradation. More notably, a marked reduction in the A260/A230 ratio (0.83-0.91) was observed in several samples (Samples 1, 2, 5, and 6). The A260/A230 ratio is highly sensitive to organic solvents, carbohydrates, and inorganic salts. Given the high salt condition (10% NaCl, ~1.7 M) used in the lysis buffer, these reduced ratios strongly point to residual NaCl carried over during ethanol precipitation. Single-step 70% ethanol rinsing may be insufficient to completely remove saturated salt precipitates when pellets are dense. Incorporating a second ethanol wash step or extending pellet drying time could effectively mitigate salt carryover. Interestingly, Samples 3 and 4 maintained acceptable A260/A230 values (2.57 and 1.82), indicating that physical manipulation during pellet washing (e.g., degree of pellet resuspension) accounts for inter-sample variability.

A striking disparity in total DNA yield was observed between male and female participants. Female urine samples yielded significantly higher DNA concentrations (up to 146.0 μg/mL) than male samples. Anatomically, female urine naturally contains a higher abundance of exfoliated squamous epithelial cells from the lower urogenital tract and vagina, whereas male urine possesses relatively low cellularity under physiological conditions. In Sample 6 (male), the total genomic DNA concentration was below the spectroscopic limit of detection (ND). Nevertheless, an A260/A280 ratio of 1.59 and an A260/A230 ratio of 0.85 were recorded. In low-concentration samples near or below the detection baseline, minor background absorbance from the buffer matrix (10 mM Tris-EDTA) or trace salt ions can distort spectrophotometric ratios.

It should be noted that the protocol proposed in this study is specialized for the extraction of cell-derived DNA from the urinary cell pellet. As the supernatant is discarded during the initial centrifugation step, the current method does not capture cfDNA. Further optimization and validation will be required to determine whether this simplified reagent system can be extended to the recovery of cfDNA from the centrifuged supernatant.

Our data revealed a marked difference in detection sensitivity depending on whether nuDNA or mtDNA was targeted. Although amplification of nuDNA failed in Sample 6 due to low cell yield, the target regions of mtDNA (HV-1 and HV-2) were successfully and reliably amplified across all samples (100% success rate). Despite the suboptimal A260/A230 ratios observed in certain samples, the downstream enzymatic compatibility remained uncompromised. Because template DNA is routinely diluted (often 1:10 to 1:50) into the final PCR reaction mixture, residual NaCl concentrations are reduced well below inhibitory thresholds for DNA polymerases (e.g., AmpliTaq Gold). This explains why amplification of high-copy mitochondrial target regions (HV-1 and HV-2) was successfully achieved even in low-yield samples such as Sample 6.

The sequence homologies for HV-1 and HV-2 were 99.4% and 97.8%, respectively, rather than 100%, which is attributed to the high degree of polymorphism and substantial inter-individual variation present in the mitochondrial D-loop region [8,12]. Whereas only two copies of nuDNA exist per cell, the mitochondrial genome is present in thousands of copies, making mtDNA an ideal clinical tracer for liquid biopsy applications.

Currently, nuDNA targets remain the primary focus of clinical genetic testing. However, tissues with high metabolic activity and continuous energy demands, such as the brain, muscle, heart, and kidneys, are known to contain high densities of mitochondria [18]. Even in healthy individuals, urine naturally contains small numbers of urothelial and squamous epithelial cells, as well as, less frequently, renal tubular epithelial cells derived directly from the kidneys [19]. One key rationale for targeting mtDNA in this study is the potential for non-invasive, early detection of mitochondrial nephropathy via urinary liquid biopsy. This condition is a rare hereditary disorder in which mutations in either mitochondrial or nuclear DNA impair cellular energy production, ultimately leading to renal dysfunction. The most common underlying cause is mtDNA abnormalities (most notably the mt.3243A>G mutation), which are frequently accompanied by extrarenal manifestations such as sensorineural hearing loss. The median time from symptom onset to genetic diagnosis can take up to six years, during which disease progression often leads to end-stage renal disease requiring dialysis or kidney transplantation [20]. Furthermore, we believe this method holds potential application for other canonical mitochondrial disorders, including mitochondrial encephalopathy, lactic acidosis, and stroke-like episodes, myoclonic epilepsy with ragged red fibers, and chronic progressive external ophthalmoplegia/Kearns-Sayre syndrome.

It should be noted that this study focuses on a DNA extraction method optimized for low-abundance cells present in the urine of healthy individuals; evaluating its applicability to pathological specimens remains a subject for future research. Furthermore, while the downstream validation in this study relied on centralized capillary sequencing, the high-yield DNA obtained via our kit-free protocol could theoretically be paired with more accessible downstream modalities. For instance, combining this extraction method with isothermal amplification techniques, such as loop-mediated isothermal amplification, or developing assay designs that rely solely on standard gel electrophoresis could eliminate the need for expensive infrastructure. Alternatively, in resource-limited settings lacking capillary sequencers, the extracted DNA is stable enough to be outsourced to commercial sequencing services, making genetic monitoring highly feasible and cost-effective. In conclusion, this method, which utilizes only two common reagents and combines a simple workflow with effective mtDNA recovery, holds potential for future application for disease monitoring using urine samples with low cell counts.

A limitation of this study is the small sample size and the lack of a detailed evaluation regarding the limit of detection using clinical disease samples. Because mitochondrial copy numbers vary widely depending on the physiological and cellular characteristics of the tissue, further validation with serially diluted samples and clinical cohorts is required to determine the exact sensitivity and efficiency of this method for future liquid biopsy applications. Additionally, a notable limitation of this study is the lack of a direct, head-to-head performance comparison with standard commercial extraction kits. Although our protocol successfully yielded sequence-ready DNA, further quantitative benchmarking against commercial column- or bead-based kits will be required in future studies to objectively evaluate comparative extraction efficiency and recovery rates.

## Conclusions

Extracting nuclear and mitochondrial DNA from scanty human urine cells is entirely feasible using standard, cost-effective reagents (10% NaCl and ProK) without specialized kit components. Because mtDNA features a significantly higher copy number per cell, it yields superior detection sensitivity compared to nuDNA in low-biomass urine specimens. While not intended for immediate clinical diagnosis, this accessible extraction strategy is highly valuable as a potential future application in non-invasive genetic testing and liquid biopsy protocols.

## References

[1] Alix-Panabières C, Pantel K (2021). Liquid biopsy: from discovery to clinical application. Cancer Discov.

[2] Oshi M, Murthy V, Takahashi H (2021). Urine as a source of liquid biopsy for cancer. Cancers (Basel).

[3] Lourenço C, Constâncio V, Henrique R, Carvalho Â, Jerónimo C (2021). Urinary extracellular vesicles as potential biomarkers for urologic cancers: an overview of current methods and advances. Cancers (Basel).

[4] Lee H, Kang SJ, Lee J, Park KH, Rhee WJ (2022). Isolation and characterization of urinary extracellular vesicles from healthy donors and patients with castration-resistant prostate cancer. Int J Mol Sci.

[5] Knowles MA (2020). FGFR3 - a central player in bladder cancer pathogenesis?. Bladder Cancer.

[6] Knowles MA, Elder PA, Williamson M, Cairns JP, Shaw ME, Law MG (1994). Allelotype of human bladder cancer. Cancer Res.

[7] Wu J, Jin S, Bai Q (2026). Noninvasive early detection and recurrence monitoring for non-muscle-invasive bladder cancer via urine tumor DNA: a prospective clinical study. MedComm (2020).

[8] Williams SB, Ye Y, Huang M (2015). Mitochondrial DNA content as risk factor for bladder cancer and its association with mitochondrial DNA polymorphisms. Cancer Prev Res (Phila).

[9] Agnoletto C, Volinia S (2022). Mitochondria dysfunction in circulating tumor cells. Front Oncol.

[10] Anderson S, Bankier AT, Barrell BG (1981). Sequence and organization of the human mitochondrial genome. Nature.

[11] Yasukawa T, Kang D (2018). An overview of mammalian mitochondrial DNA replication mechanisms. J Biochem.

[12] Holland MM, Parsons TJ (1999). Mitochondrial DNA sequence analysis - validation and use for forensic casework. Forensic Sci Rev.

[13] Cann RL, Stoneking M, Wilson AC (1987). Mitochondrial DNA and human evolution. Nature.

[14] Maitra A, Cohen Y, Gillespie SE (2004). The Human MitoChip: a high-throughput sequencing microarray for mitochondrial mutation detection. Genome Res.

[15] Jaworek H, Koudelakova V, Drabek J (2018). A head-to-head analytical comparison of Cobas 4800 HPV, PapilloCheck HPV screening, and LMNX genotyping kit HPV GP for detection of human papillomavirus DNA in cervical and cervicovaginal swabs. J Mol Diagn.

[16] Kopecká J, Matoulková D (2014). Comparison of DNA extraction methods in terms of yield, purity, long-term storage, and downstream manipulation with brewer's yeast chromosomal DNA. J Am Soc Brew Chem.

[17] (2026). National Institute of Justice. Mitochondrial DNA. https://nij.ojp.gov/nij-hosted-online-training-courses/non-str-dna-markers-snps-y-strs-lcn-and-mtdna/mitochondrial-dna-non-nuclear-dna-markers/mitochondrial-dna/applications.

[18] Rath SP, Gupta R, Todres E (2024). Mitochondrial genome copy number variation across tissues in mice and humans. Proc Natl Acad Sci U S A.

[19] Rahmoune H, Thompson PW, Ward JM, Smith CD, Hong G, Brown J (2005). Glucose transporters in human renal proximal tubular cells isolated from the urine of patients with non-insulin-dependent diabetes. Diabetes.

[20] Emma F, Bertini E, Salviati L, Montini G (2012). Renal involvement in mitochondrial cytopathies. Pediatr Nephrol.

